# Injectable Scaffold with Microfracture using the Autologous Matrix-Induced Chondrogenesis (AMIC) Technique: A Prospective Cohort Study

**DOI:** 10.5704/MOJ.2211.014

**Published:** 2022-11

**Authors:** GSY Bong, YHD Lee

**Affiliations:** 1 Department of Orthopaedic Surgery, Tan Tock Seng Hospital, Singapore; 2 Department of Orthopaedic Surgery, National University Hospital, Singapore

**Keywords:** cartilage repair, autologous matrix-induced chondrogenesis, BST-CarGel, microfracture, outcomes

## Abstract

**Introduction:**

Autologous matrix-induced chondrogenesis (AMIC) is a one-step surgical cartilage repair procedure involving the insertion of a scaffold into the chondral defect after microfracture. BST-CarGel [Smith and Nephew, Watford, England] is an injectable chitosan-based scaffold which can more easily fill defects with irregular shapes and be used to treat vertical or roof chondral lesions. The study aims to evaluate the clinical outcomes of knee cartilage repair with microfracture surgery and BST-CarGel using the AMIC technique for a minimum of two years.

**Materials and methods:**

A prospective study of patients undergoing cartilage repair with microfracture surgery and BST-CarGel at our institution from 2016 to 2019 was performed. Clinical outcomes were determined using the Lysholm Knee Scoring System and Knee Injury and Osteoarthritis Outcome Score (KOOS). These questionnaires were administered before the surgery and at a minimum of two years after surgery.

**Results:**

A total of 21 patients were identified and recruited into the study. 31 cartilage defects were seen and treated in 21 knees. These included horizontal lesions (e.g., trochlear, lateral tibial plateau), vertical lesions (e.g., medial femoral condyle, lateral femoral condyle) and inverted lesions (e.g., patella). No complications or reoperations were seen in our study population. For the average duration of follow-up of 42.5±8.55 months, there was an average improvement in Lysholm score of 25.8±18.6 and an average improvement in KOOS score of 22.5±15.0.

**Conclusion:**

BST-CarGel with microfracture surgery using the AMIC technique is a safe and effective treatment for cartilage defects in the short to medium term.

## Introduction

Chondral lesions of the knee are a common injury seen in the younger population. Widuchowski *et al* reported chondral lesions in 60% of a cohort of over 25,000 knee arthroscopies with an average age of 39 years old1. Due to the avascular, aneural, and immune-privileged nature of hyaline cartilage, the regenerative potential of cartilage after an injury is limited. The quest to repair cartilage in one-stage minimally invasive surgery is one of the holy grails in orthopaedic surgery.

Cartilage repair is recommended for symptomatic lesions in patients less than 55 years old. The techniques of cartilage repair include marrow stimulation, cell-based techniques such as autologous chondrocyte implantation, osteochondral autologous transplantation and autologous matrix-induced chondrogenesis (AMIC).

AMIC is a one-step surgical cartilage repair procedure involving the insertion of a scaffold into the chondral defect that provides a structure that mechanically stabilises the super clot and stimulates chondrogenic differentiation2. BST-CarGel [Smith and Nephew, Watford, England] is an injectable chitosan-based scaffold. Chitosan is a deacetylate derivative from chitin, which is found in exoskeletons of shellfish and insects^3^. It is biocompatible, biodegradable and shown to be a suitable scaffold for chondrocyte proliferation and differentiation^4-6^.

The study aims to evaluate the clinical outcomes of knee cartilage repair with microfracture surgery and BST-CarGel using the AMIC technique for a minimum of two years of follow-up.

## Materials and Methods

A prospective study of patients undergoing cartilage repair with microfracture fracture and BST-CarGel [Smith and Nephew, Watford, England] at our institution from 2016 to 2019 was performed. Clinical outcomes were determined using two clinically validated patient-reported questionnaires: Lysholm Knee Scoring System^7^ and Knee Injury and Osteoarthritis Outcome Score (KOOS)^8^. These questionnaires were administered before surgery and at a minimum of two years after surgery.

**Fig. 1.**
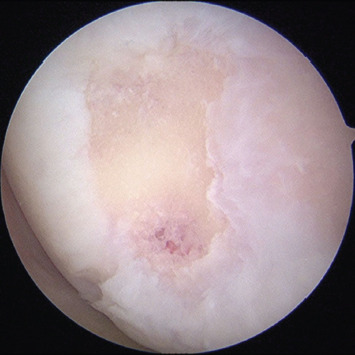
Arthroscopy image, viewing from the anterolateral portal showing medial femoral condyle full thickness cartilage defect measuring 1.5cm by 1.5cm in size.

For the operative technique, diagnostic arthroscopy of the knee joint was first performed with the use of a four-lead gravity fluid bag. Standard anterolateral and anteromedial portals were created adjacent to the patellar tendon. Using a 30° scope via the anterolateral portal, the cartilage injuries were assessed for the severity of lesion and graded according to the Outterbridge grading system, and other concomitant soft tissue injuries were inspected (Fig. 1). Where appropriate, concurrent procedures, such as meniscal repair and ligament reconstruction were performed in the same setting, before cartilage repair.

BST-CarGel was prepared according to the techniques described in the manufacturer’s product guide: The buffer solution was mixed with the chitosan solution and left for 10 minutes. A total of 5ml of autologous blood drawn via venepuncture was injected into the prepared buffer-chitosan mixture. This BST-CarGel blood-buffer-chitosan mix was drawn into an application syringe and ready to be applied to the cartilage lesion.

**Fig. 2.**
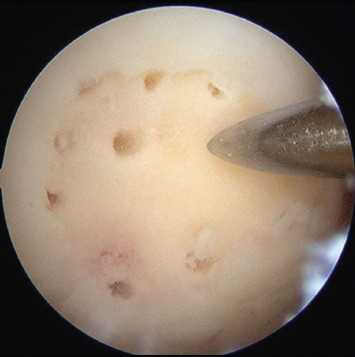
Arthroscopy image. Microfracture of the cartilage defect after preparation of the base.

The cartilage defect site was debrided using shavers and curettes. Loose chondral tissue was excised to create stable vertical walls surrounding the defect. The bed of the defect was prepared with the preservation of the subchondral plate. Microfracture was then performed with arthroscopic microfracture awls to a depth of about 4mm, with each lesion created 3 or 4mm apart throughout the surface of the cartilage defect (Fig. 2).

**Fig. 3.**
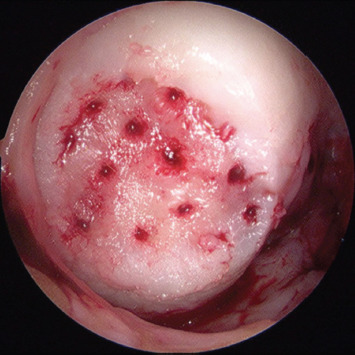
Arthroscopy image. Dry arthroscopy after completion microfracture.

**Fig. 4.**
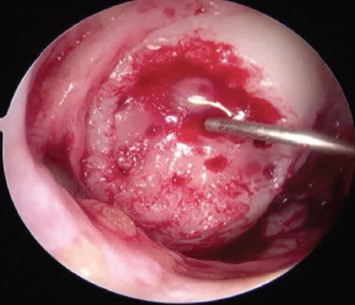
Arthroscopy image. Microfracture of the cartilage defect after preparation of the base.

**Fig. 5.**
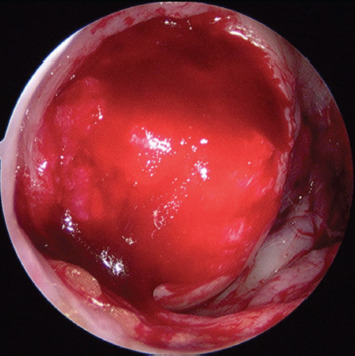
Arthroscopy image. BST-CarGel stable in cartilage defect.

The fluid was drained from the joint and dry arthroscopy was performed (Fig. 3). The knee surface and cartilage lesion were dried using suction and patties. The injectable scaffold was delivered as a viscous gel to horizontal lesions (eg. trochlear, lateral tibial plateau), vertical lesions (eg. medial femoral condyle, lateral femoral condyle) and inverted lesions (eg. patella) (Fig. 4). The scaffold was left to solidify within the defect for 5-10 minutes (Fig. 5). The knee was then mobilised, and wet arthroscopy repeated to ensure the stability of the scaffold in a fluid-filled environment.

For post-operative care, the rehabilitation range of motion protocol was dependent on the location of the lesion. For lesions of the condyles or plateau, patients were placed in a knee brace and motion restricted from 0° to 90° to limit deep flexion. For patellofemoral lesions, knee flexion was limited in the first three weeks, with the range of motion gradually increased to 90° flexion by 6 weeks. All patients were kept non-weightbearing for the first three weeks and gradually progressed to protected weightbearing (< 50% of body weight) over the next three weeks. This was to protect the microfracture clots and scaffold augmentation from dislodgement, which may occur on deep flexion or weightbearing.

Informed consent was obtained from patients before the collection of patient data. All procedures followed were in accordance with the ethical standards of the responsible committee on human experimentation (institutional or regional) and with the Helsinki Declaration of 1975, as revised in 1983.

## Results

The demographics of our study population and characteristics of cartilage lesions are summarised in Table I. A total of 21 patients were identified and recruited into the study. 85.7% were male, with an average age of 41.2 years old (range = 17-57 years old). In 65% of patients, the cartilage defect was in the right knee. An open approach was employed in five patients, with a mini-open approach used in one patient. An arthroscopic approach was used in 15 patients.

**Table I:** Demographics of study population and characteristics of lesions

Demographics	Participants (n = 21), lesions (n=31)
Age, years, mean (SD)	41.2 ( ±9.12)
Sex, male, n (%)	18 (85.7%)
Affected knee, n (%)	
Right	13 (65%)
Left	8 (35%)
Number of defects per knee, n (%)	
1	12 (57.1%)
2	8 (38.1%)
3	1 (4.76%)
Site of defect	
Trochlear	14 (45.2%)
Patellar	7 (22.6%)
Medial femoral condyle	7 (22.6%)
Lateral femoral condyle	2 (6.45%)
Lateral plateau	1 (3.26%)
Average size of defects, cm2, mean (SD)	3.36 (±3.54)
Additional diagnosis(es), n (%)	
Meniscal tear	7 (22.6%)
Anterior cruciate ligament injury	1 (13.3%)
Concurrent procedures performed in the same setting prior to cartilage repair	
Medial meniscus repair	3 (14.3%)
Lateral meniscus repair	3 (14.3%)
Anterior cruciate ligament reconstruction	1 (4.76%)
Medial patellofemoral ligament reconstruction	1 (4.76%)
Lateral patellar retinacular release	1 (4.76%)
High tibial osteotomy	1 (4.76%)

In the majority of cases (12/21, 57.1%), only one defect was present in the affected knee. Two concurrent defects were present in 8 knees (38.1%), while three concurrent defects were seen in one knee (4.8%). A total of 31 cartilage defects were seen and treated in 21 knees. The cartilage defects were most commonly seen in the trochlear (14, 45.2%), followed by the patella (7, 22.6%) and medial femoral condyle (7, 22.6%), lateral femoral condyle (2, 6.45%) and lateral tibial plateau (1, 3.26%).

The average size of each defect was 3.36 ± 3.54cm^2^ (range = 0.06-12.25cm^2^). The lateral meniscal repair was performed in the same setting on three patients. The medial meniscal repair was done on three patients, with an additional anterior cruciate ligament repair performed on one patient. Lateral patellar retinacular release, medial patellofemoral ligament repair and high tibial osteotomy were performed on one patient each. No complications or reoperations were seen in our study population.

The patient-reported outcomes are summarised in Table II. The average duration of follow-up was 42.5 ± 8.55 months (range = 28 - 58 months). There was an average improvement in Lysholm score of 25.8 ± 18.6 (range = -6 - 67), from 57.3 (range = 23 - 86) pre-operatively to 83.1 (53100) post-operatively. Similarly, the average KOOS score showed an improvement of 22.5 ± 15.0 (range = -9.7 - 55.5), from 58.4 (range = 32.1 - 85.7) pre-operatively to 80.9 postoperatively (range = 48.8 - 97.2).

**Table II:** Patient-reported outcomes

Scoring system	Average follow-up = 42.5 months (±8.55)		
	Average	Minimum	Maximum
Lysholm score			
Pre-operative	57.3	23	86
Post-operative	83.1	53	100
Difference	+25.8	-6	+67
KOOS score			
Pre-operative	58.4	32.1	85.7
Post-operative	80.9	48.8	97.2
Difference	+22.5	-9.7	+55.5

One patient reported reduced Lysholm score by 1 (preoperative score = 79; post-operative score = 78) and reduced KOOS score by 9.7 (pre-operative score = 85.7; postoperative score = 76) on 41-month follow-up. The patient underwent a concurrent lateral patellar retinacular release and had three areas of defects: 4cm^2^ trochlear defect, 0.8cm^2^ medial femoral condylar defect, 0.5cm^2^ central patellar defect. A second patient reported reduced Lysholm score by 6 (pre-operative score = 79; post-operative score = 73) on 38-month follow-up. This second patient had two areas of defects: 1cm^2^ lateral femoral condylar defect, 0.35cm^2^ trochlear defect. A 0.5cm^2^ medial femoral condylar defect with Grade 2 changes was also noted but did not undergo repair.

## Discussion

AMIC or “microfracture plus” techniques for cartilage repair are gaining popularity due to their attractiveness as a single-stage surgery. The evidence shows superior tissue repair in radiological and histological studies and improved outcomes in clinical studies^9,10^. Chondral lesions treated with the AMIC technique also had a nearly normal morphologic appearance^11^. Our study has shown that microfracture with BST-CarGel is an effective procedure in providing symptomatic relief in patients with cartilage defects in the knee.

Microfracture is the first-line option for lesions of <2.5cm^2,12^. It involves perforation of the exposed subchondral plate within the cartilage defect to form a “super clot” of marrow contents with mesenchymal stem cells to differentiate into fibrocartilage repair tissue^13^. It has been a mainstay in cartilage repair due to its effectiveness in providing symptomatic relief in the short- to medium-term^14^. However, the high fibrocartilage content in the repair tissue leads to poor long-term functional outcomes^15^ and the dislodgement of the microfracture clot may account for a lack of defect fill seen in post-operative MRIs^16^.

ACI is a two-stage procedure that harvests cartilage from regions with reduced weight-bearing requirements for expansion to chondrocytes in a lab; the chondrocytes are then implanted in the second surgery^17^. Its effectiveness lies in the ability to provide repair of the defect with more hyaline cartilage. Although it is more effective than other methods for lesions > 4cm, it is a very costly technique^12,17-19^.

AMIC is an enhancement of the microfracture technique, where the scaffold inserted into the chondral defect adds stability to the super-clot, anchoring it within the defect and reducing the risk of dislodgement^2^. In addition, scaffolds, such as chitosan used in BST-CarGel, have also been shown to promote chondrocyte differentiation^4-6^, potentially leading to increased hyaline-like cartilage repair tissue.

Several different materials have been developed for use as scaffolds. The scaffolds in the literature for AMIC include Chondro-Gide, Hyalofast, CarGel and Cartifill.

Chondro-Gide [Gesitlich Pharma, Wolhausen, Switzerland], is a solid bilayer collagen type I/III membrane made from porcine collagen^20^. Hyalofast [Anika Therapeutics, Bedford, Massachusetts, United States] is a degradable, nonwoven polymer made from a hyaluronic acid benzyl ester^21^. Hyaluronic acid has been shown to induce chondrogenesis of equine mesenchymal stem cells in the presence of autologous synovial fluid^22,23^. These solid scaffolds are effective at treating cartilage defects. A randomised controlled trial comparing AMIC using Chondro-Gide versus microfracture alone showed that AMIC had superior clinical outcomes at five years^20^. Gobbi *et al* found that cartilage repair with Hyalofast was equally as effective in patients older than 45 years old as in patients younger than 45 years old at up to 4-years follow-up^21^.

The use of solid scaffolds like Chondro-Gide and Hylofast in cartilage repair often requires additional fixation when applied to the defect, usually with fibrin glue. Whyte *et al* described a technique for the use of Hyalofast in cartilage repair. The Hyalofast solid scaffold was first cut to the appropriate size before being delivered and applied to the defect under dry arthroscopy; fibrin glue was recommended as an adjunct should more stability be required^24^. The benefit of an injectable scaffold is that it allows ease of application via arthroscopy. Being viscous in nature, these scaffolds can fill the defect with gradual solidification. They can more easily fill larger defects with irregular shapes and can be used to treat vertical or roof chondral lesions.

BST-CarGel is an injectable chitosan-based scaffold. It is mixed with autologous whole peripheral blood before injection into the defect. This scaffold-blood mixture impedes repair clot retraction and increases adhesiveness, thus stabilising the super-clot within the defect^25^. A multicentre randomised controlled trial conducted by Shive *et al* compared microfracture with BST-CarGel to microfracture alone. At five years follow-up, MRI analysis found a greater degree of lesion filling and repair tissue T2 relaxation times closer to native cartilage in the BST-CarGel group as compared to microfracture alone. Patient-reported outcomes with the Western Ontario and McMaster Universities Osteoarthritis Index (WOMAC) also showed significant improvement at five years from pre-treatment baseline^26^. Stanish *et al* compared microfracture with BST-CarGel to microfracture alone. Blinded MRI analysis showed greater lesion filling and more hyaline cartilage-like T2 values in the BST-CarGel group, while WOMAC scores showed a significant improvement at one-year follow-up. However, no significant difference was seen between the BST-CarGel group and the microfracture alone group^27^. Steinwachs *et al* retrospectively analysed pain scores, swelling scores and MRI scans in patients who were treated with microfracture and BST-CarGel, with an average of six-months follow-up. Pain and swelling scores were significantly reduced post-treatment, and post-operative MRI assessment using MOCART II scores increased significantly^28^.

Cartifill, an injectable porcine collagen scaffold, has also shown good outcomes. Kim *et al* conducted a multicentre, randomised, parallel-group trial comparing microfracture with Cartifill vs microfracture alone, looking at patient-reported outcomes at baseline, 12 months and 24 months post-intervention. There was a significantly higher odds of improvement in pain score in the Cartifill group as compared to microfracture alone. The MRI outcomes using modified MOCART, 50% defect filling and the ratio of repair tissue-to-reference cartilage were also significantly higher in the Cartifill group^29^.

Our post-operative functional outcomes are consistent with the studies on the use of enhanced microfracture techniques. Our outcomes at an average follow-up of 42.5 months postoperatively show that 90.5% (19/21) had improvement in patient-reported outcome scores. The two patients that did not have improvements had multiple cartilage lesions. This is also the first study using this scaffold in an Asian population. The option of using an injectable non-porcine derived scaffold provides us additional treatment options when treating patients who may have religious or ethical concerns about using porcine tissue. This is an important consideration in Asia.

Steinwachs *et al* described the use of BST-CarGel scaffold augmentation of a microfracture procedure with the use of dry arthroscopy. They recommended positioning the knee such that the lesion was in a horizontal position before application to avoid the gel scaffold dripping off the surface^30^. In our cohort, we have used the injectable gel scaffold in hard-to-treat positions such as vertical or even inverted lesions, so long as the injectable gel scaffold is of the right viscosity when applied. The meticulous practice of keeping the scaffold warm allows us to achieve the viscosity for stable application onto vertical or inverted lesions.

There were several limitations of our study. Firstly, as this study was conducted at a single institution by a single surgeon, only a small sample size was available. There was also a large range of defect sizes seen in our population. This, in addition to the fact that several patients underwent concurrent procedures, may have affected their reported outcomes. We were unable to stratify these factors to correlate with their outcomes due to the small sample size. However, this method enabled us to control for surgeon-dependent factors such as technical skill and familiarity with using BST-CarGel. There is potential for future studies to capture a large population size by using a standardised technique, and to differentiate outcomes based on defect size and concurrent procedures.

## Conclusion

BST-CarGel with microfracture surgery using the AMIC technique is a safe and effective treatment for cartilage defects in the short to medium term.
